# Dynamic Hydrogels Based on Double Imine Connections and Application for Delivery of Fluorouracil

**DOI:** 10.3389/fchem.2020.00739

**Published:** 2020-08-26

**Authors:** Yan Zhang, Chi-Yen Pham, Rui Yu, Eddy Petit, Suming Li, Mihail Barboiu

**Affiliations:** ^1^Key Laboratory of Carbohydrate Chemistry and Biotechnology, Ministry of Education, School of Pharmaceutical Sciences, Jiangnan University, Wuxi, China; ^2^Department of Pharmacological, Medical and Agronomical Biotechnology, Hanoi University of Science and Technology, Hanoi, Vietnam; ^3^Institut Europeen des Membranes, UMR5635, University of Montpellier, ENSCM, CNRS, Montpellier, France

**Keywords:** dynamic covalent chemistry, dynamers, hydrogels, drug delivery, imine, fluorouracil

## Abstract

Dynamic hydrogels have been prepared by cross-linking of *O*-carboxymethyl chitosan (*O*-CMCS) with reversibly connected imino-PEGylated dynamers. The double imine chitosan/dynamer and dynamer bonds and were used to provide tighter structures and adaptive drug release behaviors of the hydrogels. The structural and physical properties of the resulted hydrogels were examined, showing good thermal stability and higher swelling behaviors (up to 3,000%). When hydrogels with various composition ratios were further applied for delivery of anti-cancer drug fluorouracil (5-FU), high drug encapsulation rates were recorded, up to 97%. The release profile of 5-FU showed fast rate at the beginning, followed by slow increase to the maximum amount within 12 h, demonstrating potential as drug carriers for efficient drug delivery.

## Introduction

With the increasing interest of dynamic covalent chemistry from various fields, dynamic polymers—dynamers have presented as a powerful tool to achieve adaptive materials (Aida et al., 2012; Roy et al., 2015; Zhang and Barboiu, 2015, 2016), and found wide applications in bio-recognition (Yao et al., 2014; Yasen et al., 2017; Zhang et al., 2020b), materials design (Goor et al., 2017; Zhang et al., 2020c), drug delivery (Bakker et al., 2016), etc. The reversibly covalent bonds, such as imine, hydrazine, and disulfide, etc. have provided dynamic features to the polymers, leading to responsivity to different stimuli, including pH (Charbonneau et al., 2011), light (Fuhrmann et al., 2016), and biological targets, for example, enzymes (Zhang et al., 2016a,b, 2018b) and DNA (Catana et al., 2015; Clima et al., 2015; Zhang et al., 2018a).

As the promising drug carriers, hydrogels have been experiencing fast development in recent years (Lai and He, 2016; Oliva et al., 2017). With the retained large amounts of water and excellent biocompatibility, hydrogels demonstrated useful tool for targeted drug delivery and controlled drug release (Li and Mooney, 2016; Lei et al., 2018; Huang et al., 2020; Zhang et al., 2020a). According to the cross-linking methods, hydrogels can be formed through non-covalent physical interactions or covalent chemical bonds (Goujon et al., 2017; Gu et al., 2018). In the former case, supramolecular interactions are usually involved for the establishment of hydrogels, including hydrogen bonding, host-guest interactions, etc. (Xiao and Wang, 2018; Xiao et al., 2019; Yu J. et al., 2020). Reversible reactions, such as imine disulfide or esther bond formation, have also been applied in hydrogel preparations (Wei et al., 2015; Yamada and Schneider, 2016). Resulting in the formation of “dynamic gels” i.e., dyna-gels (Marin et al., 2014), which are dynamic on both the molecular and supramolecular levels, as systems reversibly exchanging their components, responding to external stimuli, such as pH and temperature (Deng et al., 2012). For example, pH-responsive polymers based on Schiff-base reaction have been designed and used for delivery and pseudo targeted release of doxorubicin (DOX) (Tao et al., 2018).

**Figure 1:**
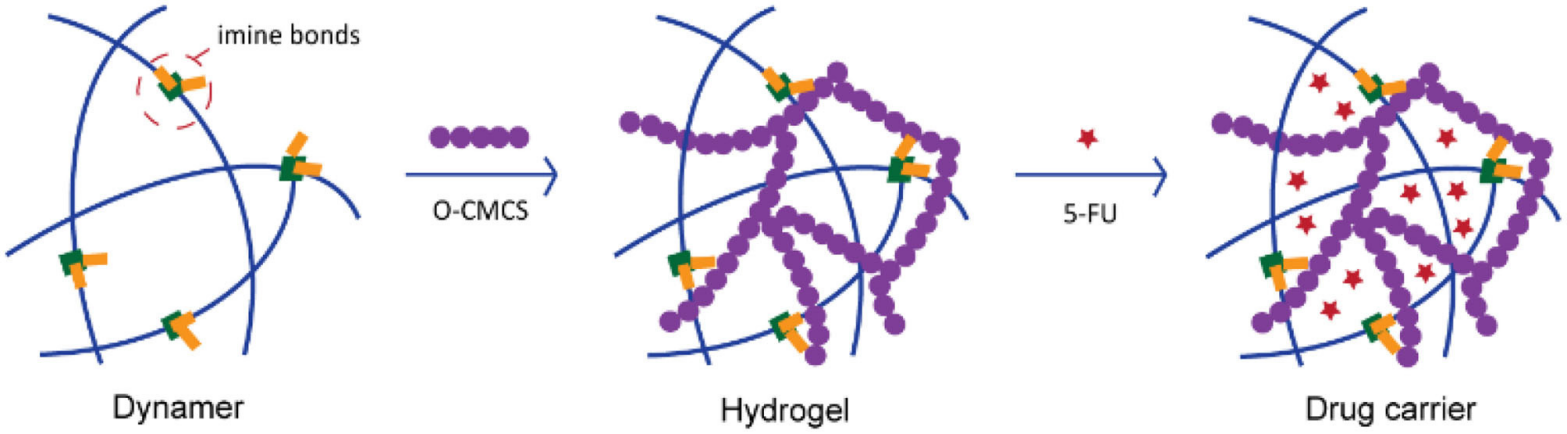
Illustration of the concept for double-imine dynamic hydrogels and application for drug loading.

Although extensive research work has been done by the insertion of dynamic reactions into hydrogel formation, dynamer as one of the starting materials, further reversibly crosslinked with natural polymers for hydrogel construction was rarely reported. Multistate pH-sensitive *O*-carboxymethyl chitosan PEGylated hydrogels, presenting outstanding self-healing properties were synthesized by our group as cell growing cyto-compatible platforms (Yu R. et al., 2020). Thus, in the current work, we would continue to explore the PEG-ylated dynameric—*O*-carboxymethyl chitosan (*O*-CMCS) cross-linked networks (Figure 1) resulting in the formation of biomimetic adaptive double imine adaptive hydrogels. These hydrogels presenting good mechanical properties and excellent swelling properties, were used as a carrier for anti-cancer drug 5-fluorouracil (5-FU) which make them beneficial for further biomedical applications.

## Materials and Methods

### Materials

1,3,5-benzenetrialdehyde was obtained from Manchester Organics, Great Britain. Poly(ethylene glycol) bis(3-aminopropyl) terminated (Mn ~ 1500) and 5-FU were purchased from Sigma Aldrich, France. *O*-carboxymethyl chitosan (*O*-CMCS) with average molecular weight (Mw) of 2 × 10^5^ was purchased from Golden-shell Biochemical Co., Ltd., China, the degree of substitution of *O*-CMCS was 80%. All organic solvents, including methanol, dichloromethane, and acetonitrile were of analytical grade, obtained from Sigma Aldrich, France.

### Synthesis of the PEGylated Dynamer

**Scheme 1:**
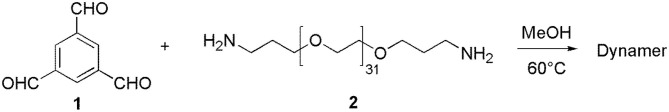
Synthesis of the PEG dynamer.

1,3,5-benzenetrialdehyde, BTA (**1**, 1 mmol, 162.14 mg), Poly(ethylene glycol)bis(3-aminopropyl) terminated (PEG, **2**, Mn~1500, 1 mmol, 1.5 g), and MeOH (30 mL) have been added in a flask, and the reaction mixture was stirred at 60°C for 3 days until equilibrium was reached (Scheme 1). After evaporation of the solvent, 20 mL of Milli-Q water were added, yielding a homogeneous dynamer solution of 50 mM (counted from the amount of BTA core structure). ^1^H NMR spectroscopy was used to monitor the formation of imine bonds. The experiments were carried out with Bruker NMR spectrometer (AMX500) of 300 MHz, by using CDCl_3_ (0.5 mL) as the solvent, while chemical shifts were recorded in ppm.

### Preparation of the Hydrogels

The gels were formed by adding *O*-CMCS to dynamer in aqueous solution. *O*-CMCS solution was prepared by adding *O*-CMCS (72.2 mg, 0.29 mmol counted from repeating unit) to 2.5 mL Milli-Q water at room temperature, followed by sonication for 15 min to yield a transparent solution of 116 mM (counted from repeating unit). Subsequently, dynamer and *O*-CMCS solutions were mixed together at r.t., with different *O*-CMCS/dynamer molar ratios of 1:4, 1:2, 1:1, 2:1, 4:1, and the resulted mixtures were kept at r.t. to afford the designed hydrogels.

### Characterization of the Hydrogels

To confirm the formation of imine bond during the gelation process, Fourier-Transform Infrared Spectroscopy (FTIR) was used to analyse the starting dynamer, *O*-CMCS solutions, and the resulted hydrogels. The experiments were performed with Nicolet Nexus FT-IR spectrometer, equipped with ATR Diamant Golden Gate.

Differential scanning calorimetry (DSC) and thermogravimetric analysis (TGA) were carried out using a DuPont instrument (TA instrument Inc., USA), to examine thermal properties of the hydrogels, under nitrogen atmosphere. Measurements for DSC were performed under a heating rate of 10°C min^−1^, with temperature range from −70 to 220°C. TGA measurements were recorded from 20 to 600°C at a heating rate of 10°C min^−1^.

### Rheological Measurements

Rheological properties of hydrogels were examined with Physical MCR 301 Rheometer (Anton Paar). The tested samples were placed on a cone plate (diameter of 4 cm, apex angle of 2°, and clearance 56 m). Measurements were made in the linear viscoelastic region.

### Swelling Test

The swelling test was performed by immersion of dried gels in Phosphate Buffered Saline (PBS) buffer (pH 7.4). The samples were taken out after 1, 3, 5, 6, 24, 48, and 168 h, wiped to remove surface water and weighed (W_s_). They were then freeze-dried for 24 h, and weighed again (W_d_). The swelling ratio was determined according to the following equation:

(1) $$\begin{array}{l} Swelling\;ratio\;\left(\%\right)\;=\;\left({\text{M}}_{\text{s}}\;-\;{\text{M}}_{\text{d}}\right)/{\text{M}}_{\text{d}}\;\times \;100 \end{array}$$

Where M_s_ and M_d_ are the mass of swollen hydrogel and of freeze dried gel, respectively.

### *In vitro* Drug Release

5-FU was selected as a hydrophilic model drug to study the *in vitro* drug release profile from the hydrogels. Firstly, 5-FU was encapsulated in hydrogels by dissolution in a dynamer solution, followed by gelation process after mixing with *O*-CMCS solution. Then the release profile was studied by immersing the hydrogel in 1 mL PBS buffer (pH 7.4) at 37°C. At each preset time interval, the whole medium was collected for measurements, and replaced by 1 mL fresh PBS solution. The released amount of 5-FU was determined by HPLC measurements.

High-performance liquid chromatography-UV (HPLC-UV) was performed by using a HPLC system equipped with Waters 717 Autosampler, Waters 616 Pump, Waters 2996 Photodiode Array Detector, and a reverse phase Thermo Scientific C18 column (L ¼ 250 mm, I.D ¼ 4.6 mm, and 5 mm particles). The mobile phase was a Buffer A (HPLC grade water 0.1% trifluoroacetic acid) and Buffer B (HPLC grade acetonitrile 0.1% trifluoroacetic acid). The flow rate was 1 mL min^−1^.

## Results and Discussions

### Characterization of the Dynamer

**Figure 2:**
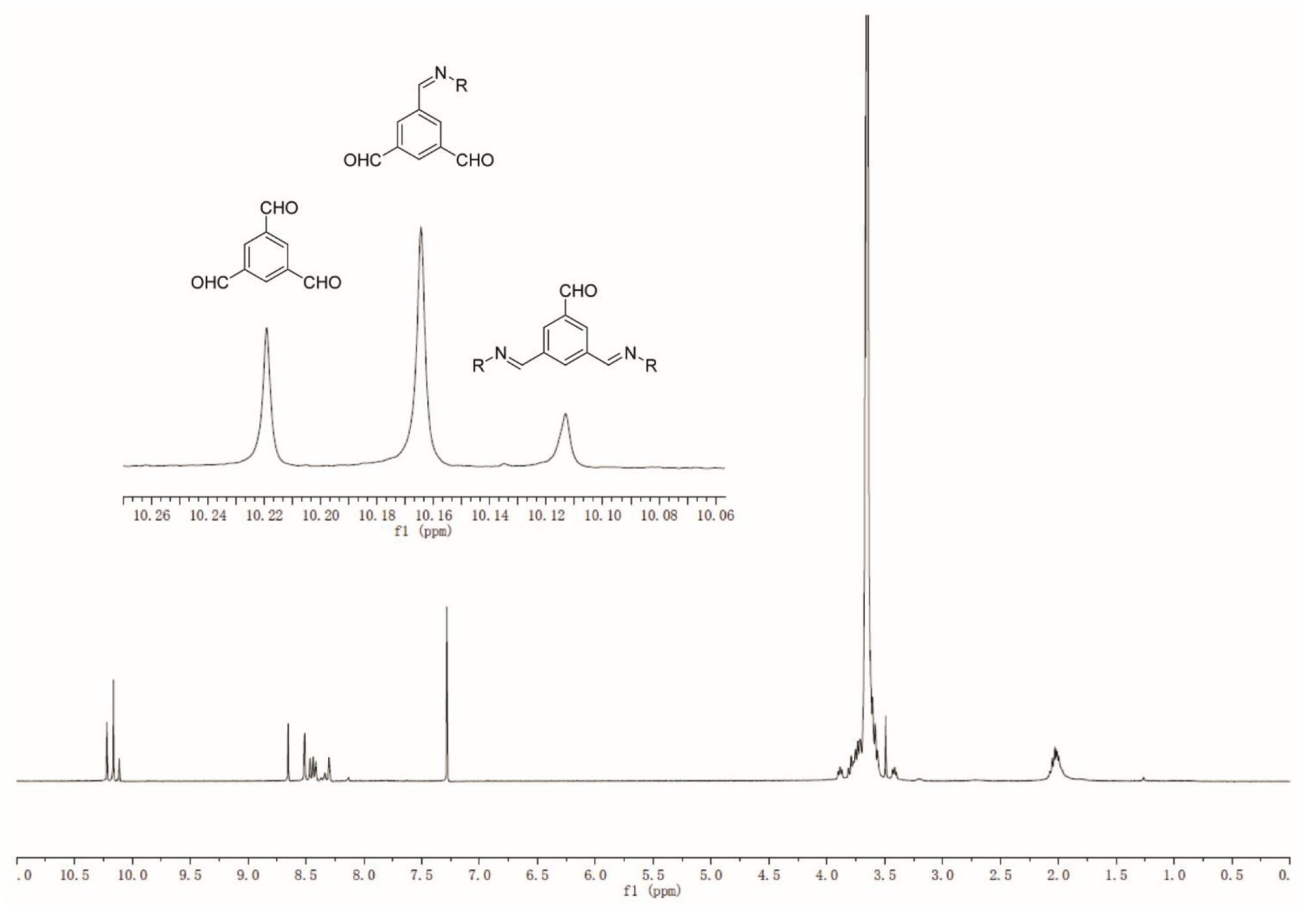
^1^H-NMR spectrum of PEGylated dynamer in CDCl_3_.

The reversible imine reaction was applied to construct a dynameric network, while using 1,3,5-benzenetrialdehyde, BTA **1** as the core structure, and the bis-amine terminated PEG **2** as the water-soluble linker (Scheme 1). The molar ratio between the two components **1** and **2** was 1:1, thus, leaving 1/3 of total aldehyde groups free to be further cross-linked to the amine groups of *O*-CMCS. After heating at 60°C for 3 days in MeOH, the equilibrium was reached as no change of the signals was observed on the ^1^H-NMR spectra. As can be seen in Figure 2, there are three aldehyde signals (10.1–10.3 ppm) which are assigned to trialdehyde **1**, mono-substituted, di-substituted aldehydes (from low to high magnetic fields). Moreover, the signals in the range of 8.0–8.7 ppm present the imine and aromatic protons. Signal at 2.0 ppm can be assigned to the methylene groups of PEG **2**.

### Characterization of the Hydrogels

**Figure 3:**
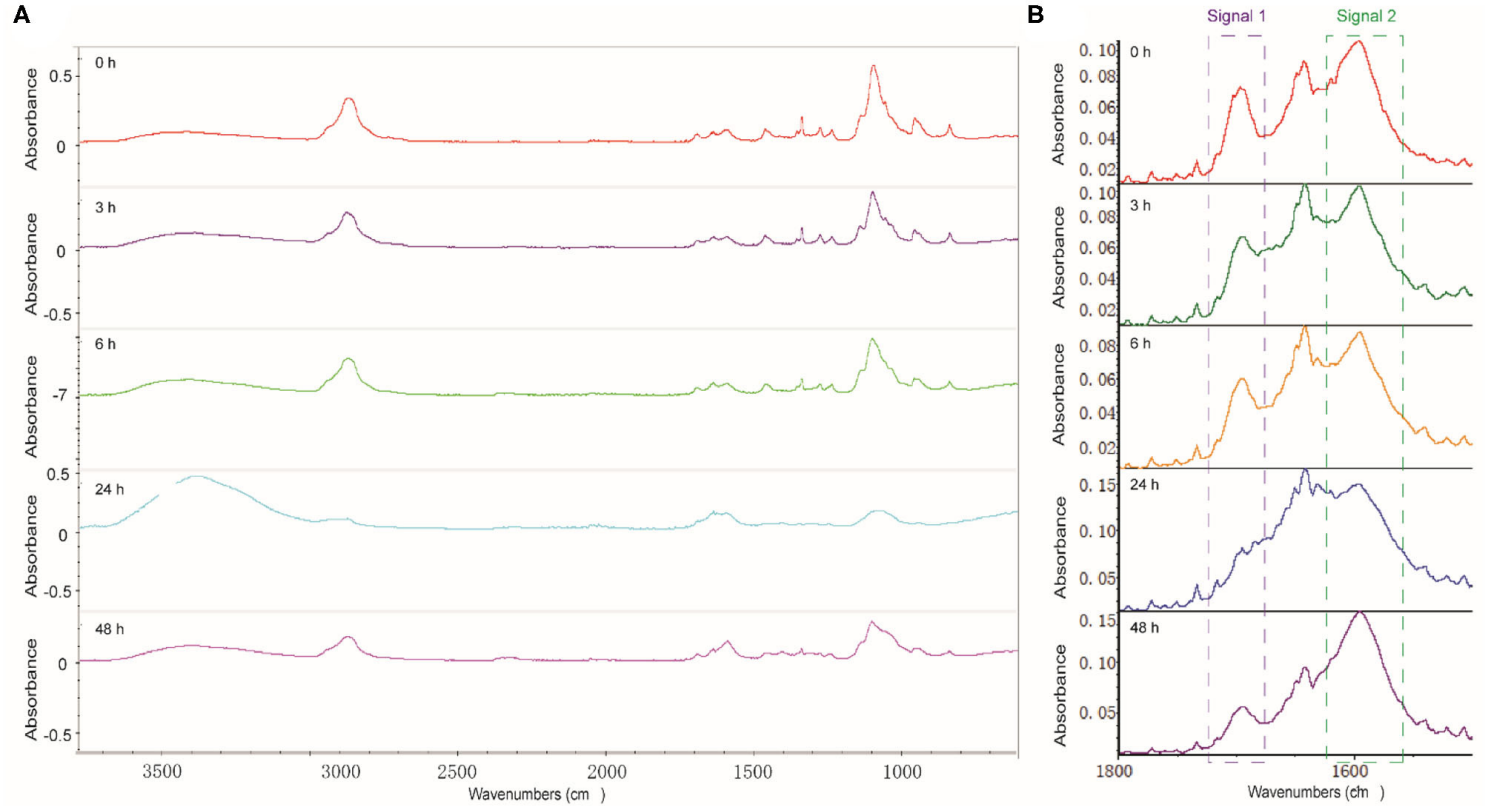
**(A)** Full scale and **(B)** enlarged carbonyl region of the FTIR spectra of hydrogels with different reaction time.

FTIR analysis was performed to figure out the degree of cross-linking between dynamer and *O*-CMCS after gelation process. The freeze-dried hydrogels after different time intervals were analyzed to follow the formation of imine bonds. As shown in Figure 3, the characteristic bands of aldehyde (signal 1) and the newly formed imine bonds (signal 2) were observed at 1700 and 1645 cm^−1^, respectively, whereas the band at 1595 cm^−1^ belongs to carboxyl groups of *O*-CMCS. With increasing time, there is significant increase of signal 2 for the imine bonds and decrease of signal 1, demonstrating higher degree of cross-linking with time.

**Figure 4:**
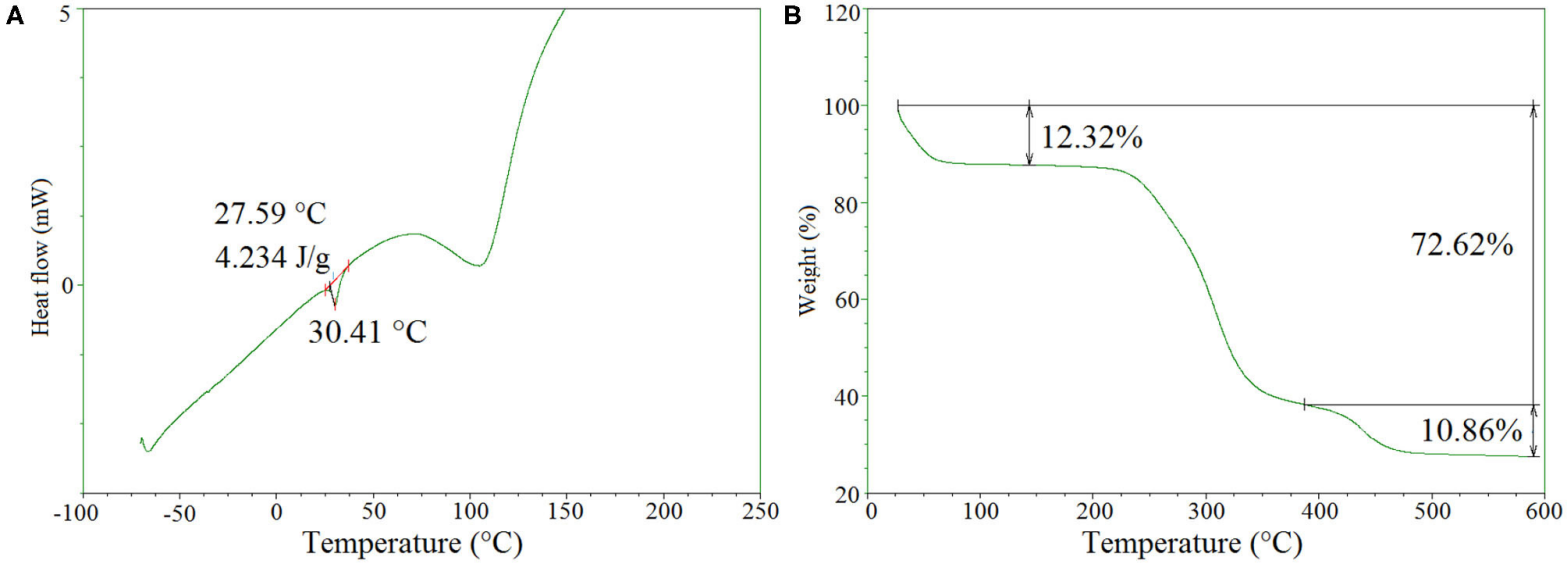
Typical **(A)** DSC and **(B)** TGA curves of the freeze-dried hydrogels.

Differential scanning calorimetry (DSC) was performed to evaluate the thermal properties of the freeze-dried hydrogels. As can be seen in Figure 4A, a large endothermic peak is detected in the range of 75–110°C due to the presence of traces of water. Moreover, a small endothermic peak was found at 30.4°C with an enthalpy of 4.2 J/g, which can be attributed to the melting of PEG. Compared to the melting temperature (Tm) of PEG1500 at 50.0°C, the decreased Tm of the hydrogels could be explained by the covalent bonding of PEG to the hydrogel network which reduced its chain mobility. From literature data, we observe only a clear water evaporation peak, reminiscent with an amorphous behavior of the *O*-CMCS (Katugampola et al., 2014).

Figure 4B presents the TGA thermogram of freeze dried *O*-CMCS-PEG hydrogels. Initially about 12.3% weight loss was detected in the range of 25–80°C due to the evaporation of loosely bound water molecules from the samples. The second stage was a predominant one, where nearly 60% weight loss occurred in the temperature range from 250 to 390°C, indicating the a onset degradation of *O*-CMCS at 240°C. At the last stage from 380 to 480°C, around 10.9% of weight loss was observed, which was assigned to the thermal degradation of PEG. It is noteworthy that nearly 27% of initial weight remains till 600°C. From the literature the O-CMCS the onset degradation occurs at 166°C, much lower that crosslinked O-CMCS hydrogels (Katugampola et al., 2014).

### Rheological Studies

**Figure 5:**
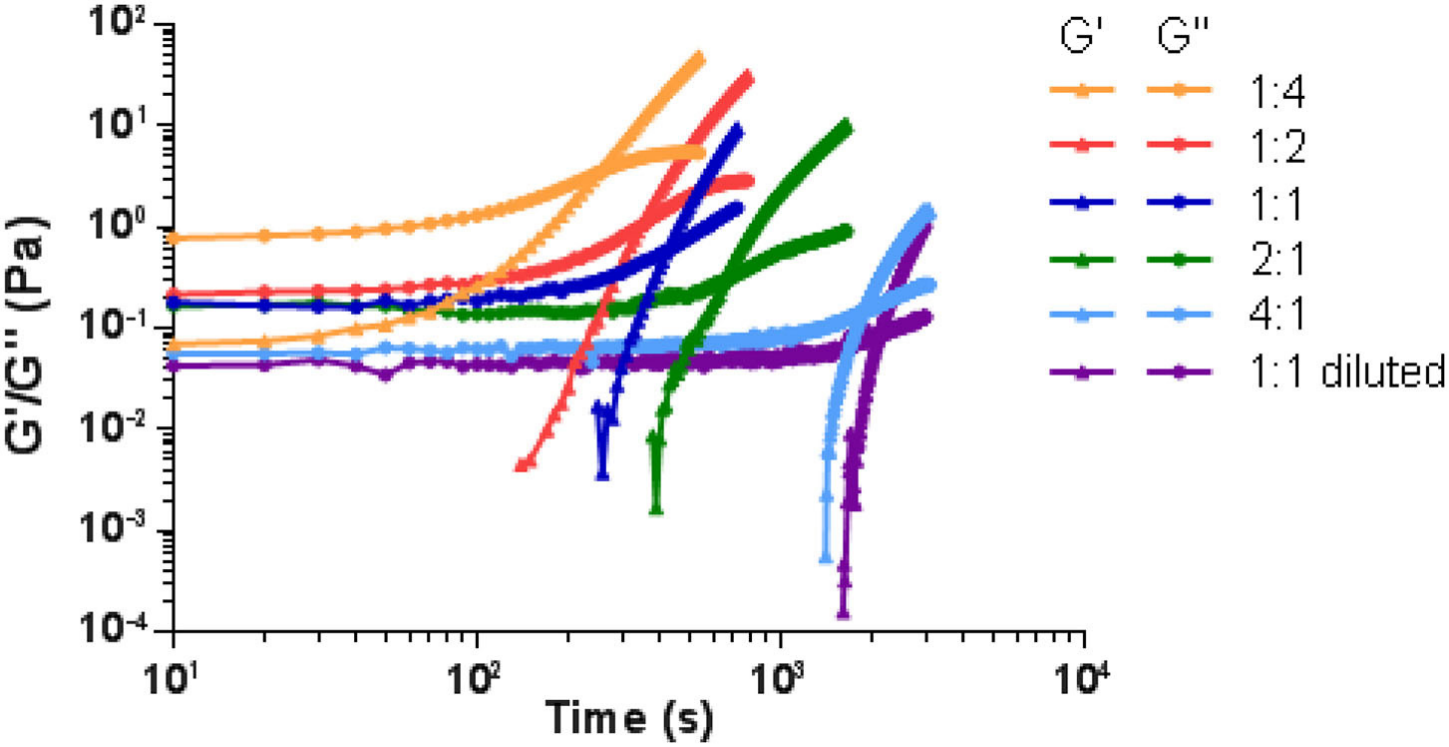
G′ and G″ variation as a function of time of the hydrogels prepared with different dynamer/*O*-CMCS molar ratios.

Rheological measurements were performed to elucidate the viscoelastic characteristics of the hydrogels. Thus, the gelation process with varying PEG dynamer/*O*-CMCS molar ratios of 4:1, 2:1, 1:1, 1:2, 1:4 was studied. Figure 5 showed the changes of storage modulus (G′) and loss modulus (G″) of various hydrogels as a function of time at 25°C. For all samples, the storage modulus was lower than the loss modulus at the beginning, which illustrated a liquid-like behavior (G′ < G″) of the starting dynamer and *O*-CMCS solutions. After an induction time, both G′ and G″ began to increase, G′ increasing at a faster rate than G″. A crossover point of G′ and G″ was detected, indicating sol-gel transition or gelation. It is noteworthy that with varying the molar ratio of PEG dynamer/*O*-CMCS from 4:1 to 1:4, higher G′ and G″ moduli shorter gelation time were obtained. In fact, the modulus at gelation point increased from 0.14 to 3.36 Pa, and the gelation time was shortened from 1,850 to 250 s. These results demonstrated that higher content of *O*-CMCS leads to increased degree of cross-linking between the aldehyde groups of cross-linking PEG dynamer and the amine groups of *O*-CMCS. Furthermore, by adopting half concentrations of both PEG dynamer and *O*-CMCS solutions at the molar ratio of 1:1 (1:1 diluted), the detected modulus at gelation point became even lower, accompanied with prolonged gelation time.

### Swelling Studies

**Figure 6:**
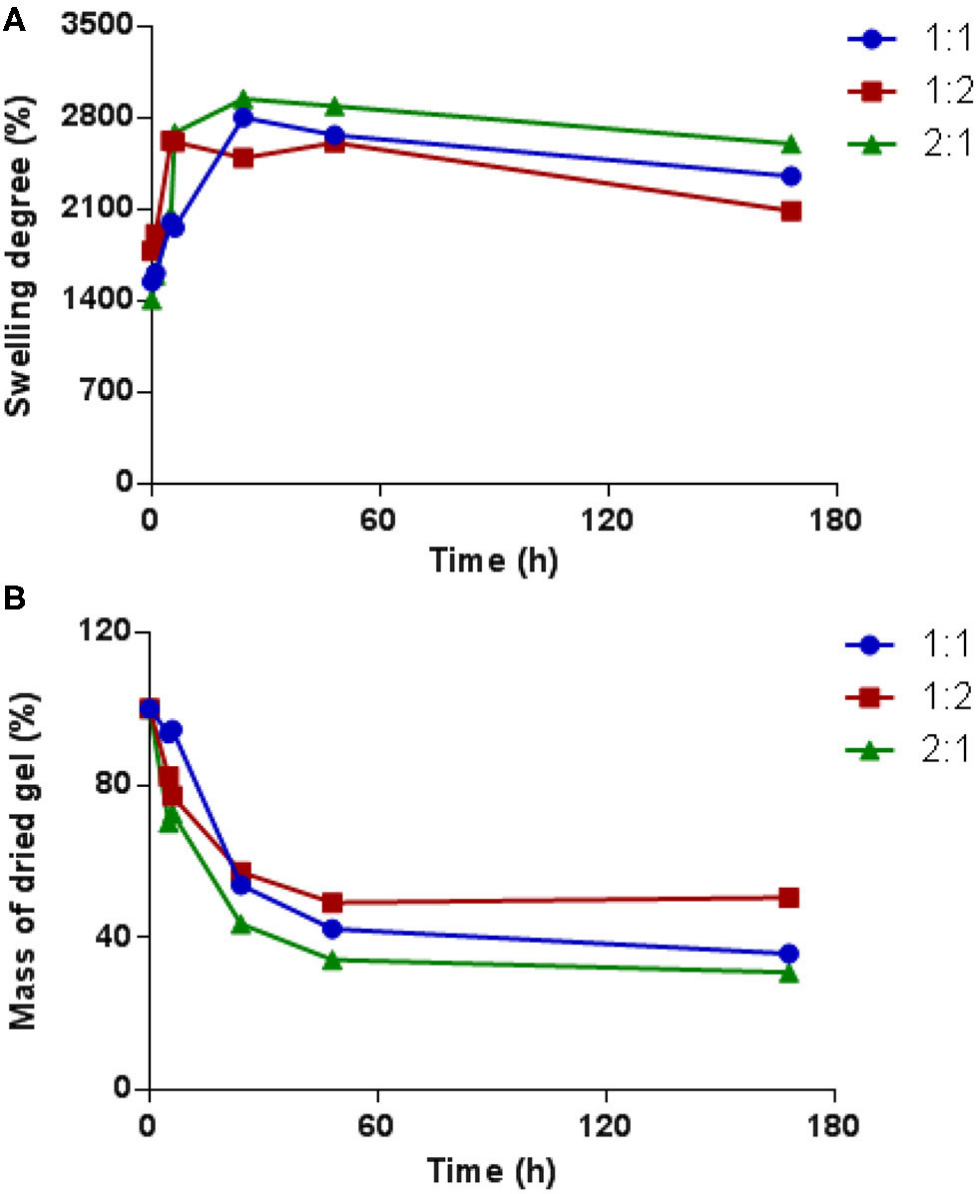
**(A)** Swelling degree variations of hydrogels obtained from different dynamer/*O*-CMCS molar ratios as a function of time; **(B)** change of dried gel masses with time.

The swelling properties of hydrogels with PEG dynamer to *O*-CMCS molar ratios of 2:1, 1:1, and 1:2 were studied in PBS at pH 7.4. As shown in Figure 6A, the swelling degree rapidly increased in the initial hours and slowly decreased thereafter. For the hydrogel formed with dynamer/*O*-CMCS molar ratio of 1:2, the highest swelling degree of 2,600% was reached after 5 h only. On the other hand, the highest swelling degrees of the hydrogels with molar ratios of 1:1 and 2:1 were reached after 24 h, with swelling degree calculated close to 3,000%. These values might be considered with a *SD*% = 8% as previously reported for similar Chitosan hydrogels (Damiri et al., 2020). These findings well agree with the less cross-linked structures of the hydrogels with molar ratios of 1:1 and 2:1 compared to the hydrogel containing more *O*-CMCS (molar ratio of 1:2). Figure 6B presents the mass changes of the freeze-dried hydrogels as a function of soaking time in PBS buffer. A rapid decrease of the hydrogel mass was observed for all the samples during the first 24 h, indicating the dissolution of non-cross-linked components of the hydrogels into the PBS. Among the different samples, the highest mass loss up to 65% was obtained at 48 h for the hydrogel with molar ratio of 2:1, which can be explained by the lowest degree of cross-linking compared to hydrogels with other molar ratios. Beyond 48 h, the mass of each hydrogel remained almost unchanged.

### *In vitro* Drug Release Performance

In the current work, 5-fluorouracil 5-FU was physically encapsulated into the dynameric hydrogels, and the *in vitro* release was studied in pH 7.4 PBS at 37°C. A calibration curve of 5-FU was first established by HPLC analysis. In all cases, high encapsulation efficiencies were obtained, up to 97%.

**Figure 7:**
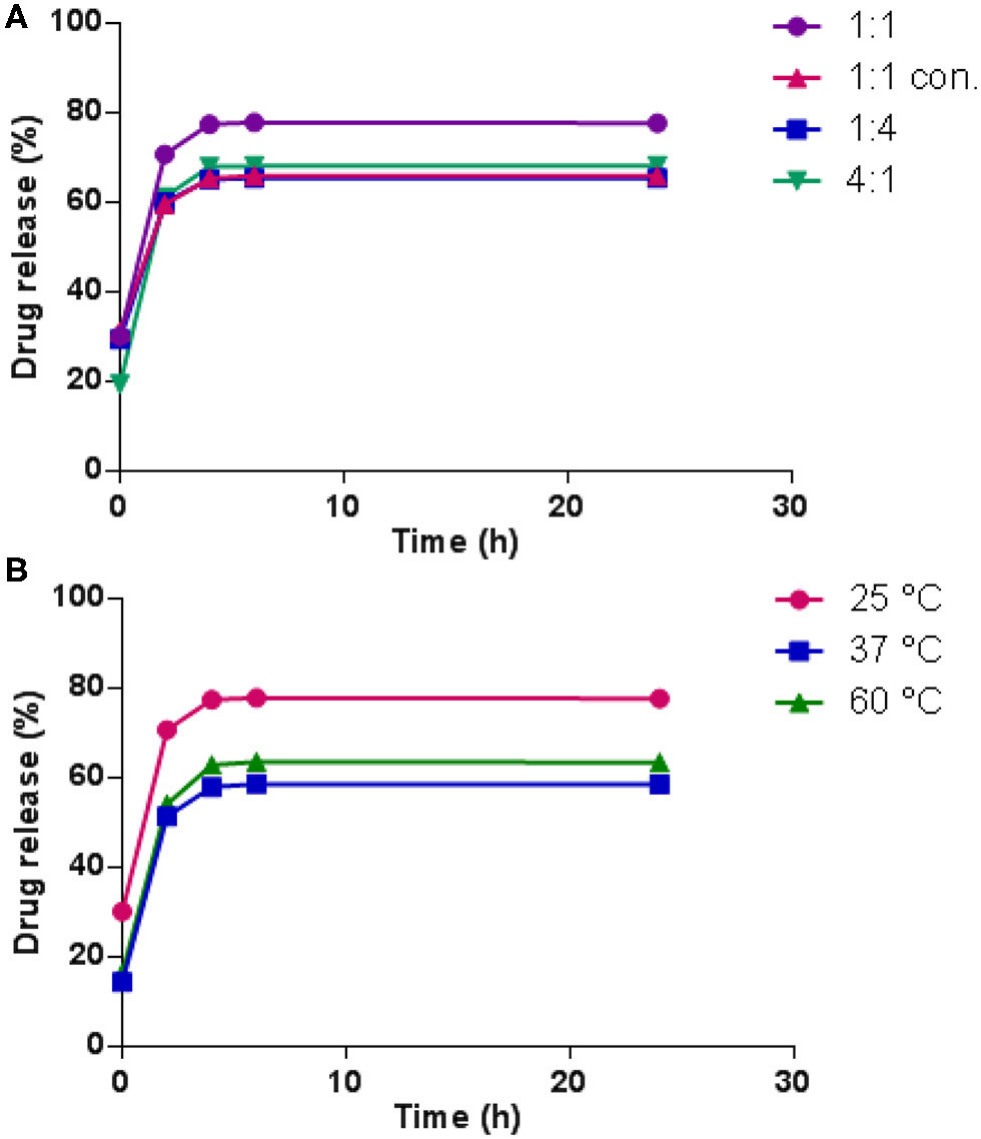
5-FU release profiles **(A)** from the hydrogels obtained from different dynamer/*O*-CMCS molar ratios at 25°C; **(B)** with different temperatures.

Figure 7A presents the release profiles of 5-FU from the hydrogels prepared at 25°C with different dynamer/*O*-CMCS molar ratios. The drug release values might be considered with a *SD*% = 5% as previously reported for similar Chitosan hydrogels (Damiri et al., 2020). For the hydrogel made from molar ratio of 1:1, the release rate of 5-FU appeared very fast at the beginning, reaching 71% after 2 h, followed by slow increase with total release of 77% by 24 h. Comparatively, hydrogels made from molar ratio of 1:4 and twice concentrated starting materials exhibited a total release of 5-FU below 70%, probably due to the highly cross-linked structures of the hydrogels. However, the hydrogel with molar ratio of 4:1 and thus less cross-linked structure, also showed lower total release of 5-FU compared to the hydrogel made from molar ratio of 1:1. It could be possibly explained by the high affinity between hydrophilic PEG fragments and 5-FU. On the other hand, in all cases, drug release leveled off beyond 5 h, when the volume shrinkage of the hydrogels was observed, indicating the tighter entrapment of 5-FU inside the hydrogel structure.

To examine the effect of gelation temperature on the release profile of 5-FU from the hydrogels, hydrogels prepared at 37 and 60°C were also tested. As illustrated in Figure 7B, much lower initial release was observed by elevating the gelation temperature, 14% at 37°C and 16% at 60°C compared to 30% at 25°C. Meanwhile, the total release of 5-FU also decreased to a large extent at higher temperatures, which confirmed the lower drug release with highly cross-linked hydrogel structures.

## Conclusions

In the current work, dynamic hydrogels were prepared cross-linking of PEG dynamers with *O*-carboxymethyl chitosan *via* amino/carbonyl-imine chemistry. The hydrogels were characterized by NMR, FT-IR spectroscopies, demonstrating the complete formation of the cross-linked imine bond networks of the hydrogel. The swelling property of the hydrogels was subsequently examined. Rheological measurements revealed that higher amount of *O*-CMCS contributes to shorter gelation time due to more cross-linking. Thereafter, they were successfully applied for delivery of anti-tumor drug 5-FU. The loading of 5-FU was efficient, with encapsulation rate up to 97%. The release profile was fast at the beginning, then gradually reached the maximum level within half a day. This is the first example of dynameric hydrogels constructed through cross-linking of imine bonds, revealing promising drug delivery properties. Further work on stimuli-responsive dynamic hydrogels for controlled drug delivery system is under study.

## Data Availability Statement

The raw data supporting the conclusions of this article will be made available by the authors, without undue reservation.

## Author Contributions

Conceptualization: MB and SL. Synthesis and characterization of materials: YZ, C-YP, RY, and EP. Writing—original draft preparation: YZ. Supervision: MB and SL. Funding acquisition: MB. All authors contributed to the article and approved the submitted version.

## Conflict of Interest

The authors declare that the research was conducted in the absence of any commercial or financial relationships that could be construed as a potential conflict of interest.
